# Safety and pharmacological characterization of the molecular tweezer CLR01 – a broad-spectrum inhibitor of amyloid proteins’ toxicity

**DOI:** 10.1186/2050-6511-15-23

**Published:** 2014-04-16

**Authors:** Aida Attar, Wai-Ting Coco Chan, Frank-Gerrit Klärner, Thomas Schrader, Gal Bitan

**Affiliations:** 1 Department of Neurology, David Geffen School of Medicine, University of California at Los Angeles, Los Angeles, CA 90095-7334, USA; 2 Brain Research Institute, University of California at Los Angeles, Los Angeles, CA 90095, USA; 3 Molecular Biology Institute, University of California at Los Angeles, Los Angeles, CA 90095, USA; 4 Institute of Organic Chemistry, University of Duisburg-Essen, Essen 45117, Germany

**Keywords:** Molecular tweezers, Toxicity, Alzheimer’s disease, Drug development, Blood–brain barrier

## Abstract

**Background:**

The “molecular tweezer” CLR01 is a broad-spectrum inhibitor of abnormal protein self-assembly, which acts by binding selectively to Lys residues. CLR01 has been tested in several *in vitro* and *in vivo* models of amyloidoses all without signs of toxicity. With the goal of developing CLR01 as a therapeutic drug for Alzheimer’s disease and other amyloidoses, here we studied its safety and pharmacokinetics.

**Methods:**

Toxicity studies were performed in 2-m old wild-type mice. Toxicity was evaluated by serum chemical analysis, histopathology analysis, and qualitative behavioral analysis. Brain penetration studies were performed using radiolabeled CLR01 in both wild-type mice and a transgenic mouse model of Alzheimer’s disease at 2-m, 12-m, and 22-m of age. Brain levels were measured from 0.5 - 72 h post administration.

**Results:**

Examination of CLR01’s effect on tubulin polymerization, representing normal protein assembly, showed disruption of the process only when 55-fold excess CLR01 was used, supporting the compound’s putative “process-specific” mechanism of action.

A single-injection of 100 mg/kg CLR01 in mice – 2,500-fold higher than the efficacious dose reported previously, induced temporary distress and liver injury, but no mortality. Daily injection of doses up to 10 mg/kg did not produce any signs of toxicity, suggesting a high safety margin.

The brain penetration of CLR01 was found to be 1 - 3% of blood levels depending on age. Though CLR01 was almost completely removed from the blood by 8 h, unexpectedly, brain levels of CLR01 remained steady over 72 h.

**Conclusion:**

Estimation of brain levels compared to amyloid β-protein concentrations reported previously suggest that the stoichiometry obtained *in vitro* and *in vivo* is similar, supporting the mechanism of action of CLR01.

The favorable safety margin of CLR01, together with efficacy shown in multiple animal models, support further development of CLR01 as a disease-modifying agent for amyloidoses.

## Background

Alzheimer’s disease (AD) along with over 30 other diseases, are amyloidoses, in which aberrant protein folding and aggregation is a central pathologic process. Amyloidoses are characterized by self-assembly of one or more proteins into toxic oligomers and insoluble amyloid. Currently, amyloidoses have no cure. Inhibition of the aberrant aggregation process is highly challenging because unlike traditional drug targets that have defined structures and in many cases, specific binding sites or active sites, toxic oligomers of amyloidogenic proteins are metastable structures that sample numerous conformations and amyloid fibrils are characterized by flat surfaces. These structures largely are devoid of specific binding pockets [1,2]. A possible solution to these challenges is to aim for one step prior to the unknown and unfavorable structures, specifically targeting the aberrantly self-associating proteins at the level of amino acid interactions.

**Figure 1:**
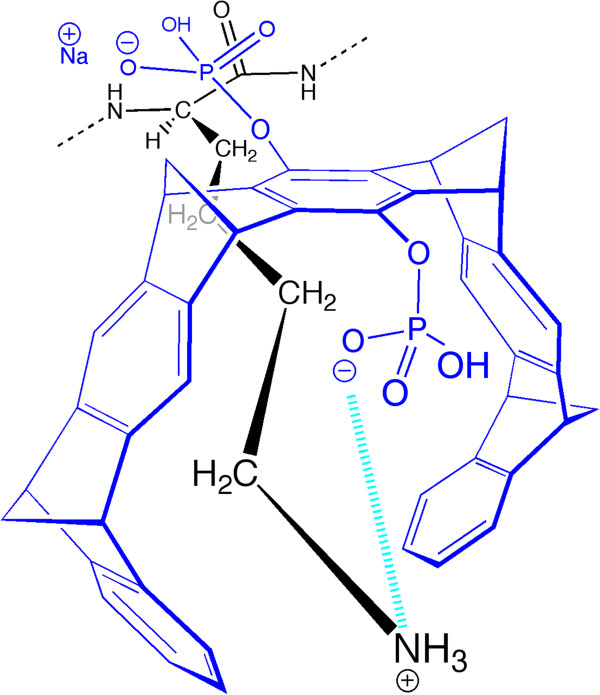
**Schematic representation of the interaction between CLR01 and Lys.** CLR01 is depicted in blue and Lys in black. The Coulombic attraction between a negatively charged phosphate group at the bridgehead of CLR01 and the positively charged ϵ-NH_3_^+^ group of Lys is illustrated in cyan. The binding is stabilized also by hydrophobic interaction between the hydrocarbon side arms of CLR01 and the butylene chain of the Lys.

Recently, we reported that the molecular tweezer, CLR01, is a novel, broad-spectrum inhibitor of abnormal protein self-assembly, which acts by a “process-specific” mechanism and inhibits the aggregation and toxicity of multiple amyloidogenic proteins [3-5]. CLR01 is a small molecule, originally developed as an artificial Lys receptor [6,7] that binds Lys residues with low micromolar affinity [3,6] or in certain cases, sub-micromolar affinity [8]. The binding is highly labile [9], yet it is selective to Lys and involves inclusion of the Lys side-chain within the tweezer cavity (Figure 1). CLR01 also binds to Arg with ~10-fold lower affinity [7,10]. Selective binding to Lys is achieved by a combination of hydrophobic and electrostatic interactions. Lys is the only proteinaceous amino acid side-chain that effectively forms both types of interactions – hydrophobic interactions involving the butylene chain, and Coulombic attraction/repulsion of its ϵ-NH_3_^+^ group. Both types of interactions are important in aberrant protein self-assembly. Thus, CLR01 competes for the same interactions that are key to nucleation and aggregation by most amyloidogenic proteins [11,12].

The moderate-affinity binding of CLR01 to Lys is key to its process-specific mechanism. Unlike the forces that mediate normal protein biology, those that control the abnormal assembly of amyloidogenic proteins were not optimized by evolution. Consequently, the binding energies involved are substantially weaker than those controlling normal protein structure and function. Therefore, although CLR01 may bind to exposed Lys residues in virtually any protein, we reasoned that at sufficiently low concentrations, labile binding with micromolar affinity would only affect relatively weak interactions, such as those that mediate aberrant protein oligomerization and nucleation.

The data generated to date have supported our conjecture. *In vitro* studies of metabolic toxicity and drug–drug interaction involving the cytochrome P450 system showed minimal inhibition of five major isoforms with half-maximal inhibition concentration values above levels expected to cause drug–drug interactions [5]. Minimal activation of the cytochrome P450 system by CLR01 was detected up to 10-μM concentrations in a cell-culture system compared to the antibiotic rifampicin, which was used as a positive control [5]. In nerve growth factor-differentiated rat pheochromocytoma cells treated with CLR01, no toxicity was detected up to 200 μM, whereas a mild decrease in cell viability was observed at 400 μM—1 - 3 orders of magnitude higher than concentrations needed for inhibition of the toxicity of different amyloidogenic proteins in cell culture [3,13].

*In vivo*, CLR01 prevented deformation and mortality in a zebrafish model of α-synuclein (α-syn) toxicity by keeping α-syn soluble, preventing its neurotoxic effects, and promoting disinhibition of the 26S ubiquitin-proteasome system, thus allowing it to degrade the excess α-syn [4]. Peripheral, subcutaneous (SC) administration of CLR01 in a triple-transgenic (3×Tg) mouse model of AD [14] resulted in a significant decrease in amyloid plaque burden and hyperphosphorylated tau, with an accompanying decrease in microgliosis [5]. Similarly, peripheral administration of CLR01 in a mouse model of familial amyloidotic polyneuropathy expressing mutant transthyretin led to a significant decrease in transthyretin deposition and associated endoplasmic-reticulum stress, apoptosis, and protein oxidation markers [15]. In support of the putative process-specific mechanism of CLR01, no signs of toxicity were observed in any of these studies. CLR01 was used at up to 10 μM in the zebrafish model (in the water environment [4]), at 40 μg/kg/day in the AD mouse model [5], and at 1.2 mg/kg/day in the transthyretin model [15].

Further support for the proposed process-specific mechanism came from the observation that CLR01 did not affect processing of amyloid β-protein precursor (APP) in the treated AD mice. In APP, Lys residues are located N-terminally to the α- and β-secretase cleavage sites. Ostensibly, CLR01 binding to these residues could have affected APP processing. However, no differences were found in levels of APP cleavage products between brain extracts of vehicle- or CLR01-treated mice [5]. To further examine the putative process-specific mechanism and toxicity profile of CLR01, here we evaluated the effect of the compound *in vitro* on a physiologic (as opposed to aberrant) protein self-assembly process—tubulin polymerization—and *in vivo* using wild-type (WT) mice to which CLR01 was administered at high doses either as a one-time bolus or daily for 1 month.

A large number of amyloidoses affect the central nervous system (CNS). If molecular tweezers are to be developed as drugs for these diseases, they likely will need to cross the blood–brain barrier (BBB). In the AD-mouse-treatment study, SC administration of CLR01 resulted in clear CNS effects [5], suggesting that the compound penetrated through the BBB into the brain of the mice. However, in that study we only began to measure the brain penetration levels and did not address the effect of age or disease. The BBB becomes compromised with aging [16] and this compromise is thought to be exacerbated in patients with certain neurodegenerative diseases, including AD [17-19]. Previously, using ^3^H-CLR01 injected intravenously, we found radioactivity levels in the brain to be ~2% of blood levels in 12-m old WT and 3×Tg AD mice [5]. We present here a characterization of the BBB’s permeability to CLR01 and the effects of age and presence of AD-linked transgenes. We also assess a likely route of metabolism of CLR01 in mouse brain.

## Methods

### Mice

All procedures were compliant with the National Research Council Guide for the Care and Use of Laboratory Animals, and approved by the University of California at Los Angeles (UCLA) Institutional Animal Care Use Committee. Two-month old WT C57BL/6J mice for toxicity studies were purchased from Jackson Laboratory (Bar Harbor, Maine, Stock 000664). 3×Tg and WT mice with the same genetic background [14] for BBB studies were bred at UCLA. Mice were housed 2–4 per cage under standard conditions and maintained on a 12-h dark and 12-h light cycle with *ad libitum* access to rodent chow and water.

### CLR01

CLR01 was produced and purified as described previously [7]. ^3^H-CLR01 was prepared by Moravek Biochemicals (Brea, CA) using a method that provides ^3^H incorporation into the hydrocarbon skeleton (i.e., non-labile protons) [20] yielding pure ^3^H-CLR01 with specific activity 1.3 Ci/mmol.

### Inhibition of tubulin polymerization

The effect of CLR01 on tubulin polymerization [21,22] was analyzed using a commercial kit (Cytoskeleton, Inc., Denver, Colorado). Three mg/ml porcine brain tubulin (~18 μM) were allowed to polymerize at 37 ºC in the absence or presence of CLR01 concentrations ranging from 10–1,000 μM. The turbidity of the solution was measured as absorbance at λ = 340 nm using a Synergy HT microplate reader (BioTek, Winooski, VT). The data are an average of three independent experiments with two wells per condition.

### Toxicity evaluation

For acute-toxicity studies, 2-m old C57BL/6J mice were administered saline-vehicle, 10 mg/kg, or 100 mg/kg CLR01 by a single intraperitoneal (IP) injection. The mice were sacrificed 24-h after the injection. For chronic toxicity studies, 2-m old C57BL/6J mice were administered saline-vehicle, 3 mg/kg, or 10 mg/kg CLR01 by daily IP injection for 30 days. Acute-study mice were visually monitored for 1 h after injection and then every 50 min for 10 min over the first 6 h of the experiment for changes in activity and behavior. The mice also were monitored every 110 min for 10 min during the last 6 h of the experiment until they were sacrificed. Chronic-study mice were monitored for 1 h after injection and then 3 times a day for 10 min each, every day of the first week. During that week there were no appreciable changes in the behavior, appearance, or weight of the mice. Therefore, monitoring was reduced to twice a day during the remainder of the experiment. On all occasions, the mice were monitored for any signs of severe toxicity, including bruising or bleeding, pale mucous membranes or extremities, diarrhea, dehydration, neurological signs, such as difficulty ambulating or paralysis, tachypnea or dyspnea, or abdominal distension.

Following the treatment, mice were anesthetized with pentobarbital and blood was collected by cardiac puncture and placed in tubes containing a clot activator for serum separation (Capiject T-MG tubes, Terumo Medical Products, Somerset, NJ). Then, the lungs were filled through the trachea with 4% paraformaldehyde to prevent collapse, and tissues (brain, heart-lung, liver, kidney, and spleen) were collected and fixed for 72 h in 4% paraformaldehyde at a ratio of ~1:10 tissue:fixative (v/v). Tissues then were transferred into a 70%-ethanol solution and transferred to the UCLA Mouse Pathology Core for paraffin embedding, sectioning, and tissue histopathology analysis. Serum was analyzed by the UCLA Division of Laboratory Animal Medicine (DLAM) Animal Serology & Molecular Diagnostic Laboratory for an 11-panel serum chemical analysis using the ACE Alera Clinical Chemistry system (Alfa Wassermann Diagnostic Technologies, West Caldwell, NJ). The panel included: alanine aminotransferase, aspartate aminotransferase, albumin, alkaline phosphatase, creatinine, total bilirubin, lactate dehydrogenase, blood urea nitrogen, cholesterol, total protein, and glucose.

### Plasma concentration and blood–brain barrier permeability

For studies of plasma concentration, CLR01 was administered by either SC or intravenous (IV) injection at 1 mg/kg, or by oral gavage at 10 mg/kg, and plasma was collected at time points between 0.33 - 24 h. Three mice were used per time point. The concentration of CLR01 in plasma was determined by Wolfe Laboratories Inc. (Watertown, MA) using liquid chromatography-mass spectrometry (LC-MS) by interpolation of sample peak area data into the calibration curve.

The following groups of mice were used for CLR01 BBB penetration studies: 3×Tg and the corresponding WT mice at 2-m, 12-m, and 22 - 24-m (hereafter referred to as 22-m) of age. The groups were: 2-m WT, 2-m Tg, 12-m WT, 12-m Tg, 22-m WT, 22-m Tg. Mice were administered ^3^H-CLR01 intravenously. Two μCi per gram of mouse body weight, which are equal to 11.86 μg/g of CLR01 in which ^3^H-CLR01 made up 10% of the total CLR01, were injected into the jugular vein. Blood and brain were collected at 0.5, 1, 3, 8, 24, or 72 h (not all time points were collected for all groups, see the Results section). For times ≤ 3 h, mice were anesthetized by IP injection of ketamine and xylazine. The mice remained anesthetized following the injection until the specified time point, at which point they were given a lethal dose of pentobarbital. Then, blood was collected via a cardiac puncture, and the brain harvested with or without a perfusion step Pharmacokinetics of CLR01 *in vivo*. For time points 8–72 h, mice were not anesthetized and ^3^H-CLR01 was injected into the tail vein. This change was due to the difficulty of keeping mice anesthetized for longer than 3 h. No differences were observed between mice given anesthesia and jugular vein injections and mice receiving tail vein injections.

Euthanasia procedures were the same as described above. For all mice, one hemisphere of the brain and 100–350 μl of blood were separately digested following instructions from Perkin-Elmer (document: Scintillation Cocktails and Consumables) with 1 ml Solvable (Perkin-Elmer, Waltham, MA), added to Ultima Gold Liquid Scintillation Cocktail (Perkin-Elmer) and read in a Triathler Liquid Scintillation Counter model 425–034, (Hidex, Turku, Finland). Brain permeability percentage was calculated as counts per minute (CPM) per g of brain relative to CPM per ml of blood. The data are an average of values from three mice per genotype/age/time combination.

For CLR01 transport-saturation studies using 5× the CLR01 dose, ^3^H-CLR01 was kept at 10% of the total CLR01 mixture and a total of 59.3 μg of CLR01 (10 μCi) per g of mouse body weight was injected IV (22-m WT 5× dose). For CLR01 brain-accumulation studies, two 11.86-μg/g injections were administered at equal time intervals (22-m WT 2× inj). In these experiments, mice were injected at time = 0 and at t = ½ of euthanasia time. For example, in the original, single-injection experiments, a mouse would receive an injection at t = 0 and then be euthanized at t = 1 h. In this experiment, a mouse received one injection at t = 0, a second injection at t = 0.5 h, and then was euthanized at t = 1 h.

In experiments using ^3^H-CLR01, urine was collected when possible over the period between injection and euthanasia. In all cases, we found that the urine was radioactive. However, comparison among mice proved to be difficult. We could not normalize the radioactivity because the amount of urine in the bladder prior to injection and the volume produced during the experiment could not be calculated. Thus, we can simply conclude qualitatively that CLR01 is excreted through the urine, but cannot provide quantitative measures of what percentage of the compound is excreted this way.

### *In vitro* metabolism

Potential dephosphorylation of CLR01 was analyzed by incubating 100 nmol CLR01 with 0.08 units of alkaline phosphatase (ALP; calf intestinal alkaline phosphatase, Promega, Madison, Wisconsin) for 60 min at 60°C. One enzymatic unit is defined as the amount of enzyme required for catalyzing the hydrolysis of 1 μmol of *p*-nitrophenylphosphate per minute. *p*-Nitrophenylphosphate disodium salt at 5–50 nmol (Fisher, Waltham, Massachusetts) was used for generation of a standard curve. The amount of inorganic phosphate generated was measured spectrophotometrically using an EnzChek Phosphate Assay kit (Life Technologies, Carlsbad, California) according to the manufacturer’s instructions on a DU-640 spectrophotometer (Beckman Coulter, Brea, California) at λ = 360 nm. Baseline values were subtracted from readings and compared to the standard curve resulting from serial ALP reactions to calculate the amount of inorganic phosphate. Similarly, potential dephosphorylation of CLR01 by brain homogenates was measured. For these experiments, one brain hemisphere was homogenized by sonication in the presence of cOmplete protease-inhibitor cocktail (Roche, Penzberg, Germany). Protein concentration was measured using a BCA Protein Assay Kit (Pierce, Rockford, Illinois). A “phosphate-mop” system was used according to the EnzChek Phosphate Assay kit instructions to sequester inorganic phosphates naturally present in 1.5 mg of brain, and then 50-nmol CLR01 or different concentrations of *p*-nitrophenylphosphate disodium salt were added and incubated for 60 min at 60°C.

### Statistics

Data are shown as mean ± SD or mean ± SEM as appropriate. Statistical analysis was performed using Prism 6.0c (GraphPad, La Jolla, CA). For all experiments, 2-way analysis of variance followed by Sidak’s multiple comparisons test *post-hoc* analysis were used. The level of significance was set at *p* < 0.05.

## Results

### *In vitro* examination of the process-specific mechanism of CLR01

As stated above, the mechanism by which CLR01 remodels the assembly of amyloidogenic proteins into non-toxic assemblies that can be degraded by normal clearance mechanisms is by its specific binding to Lys residues. The mechanism is “process-specific” because it is postulated to affect only the aberrant assembly of proteins that leads to toxic oligomers and aggregates, but not normal protein structure, function, or assembly as happens, e.g., in tubulin polymerization. To test whether this indeed is the case, we examined the effect of CLR01 on tubulin polymerization [21,22]. Three mg/ml (~18 μM) porcine brain tubulin, which contains 3.8% Lys and 4.8% Arg, was allowed to polymerize in the absence or presence of CLR01 concentrations ranging from 10–1,000 μM.

**Figure 2:**
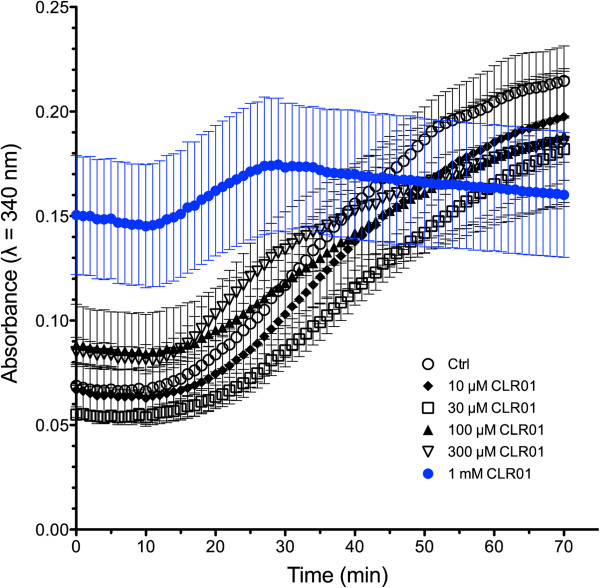
**Impact of CLR01 on tubulin polymerization.** Tubulin was allowed to polymerize in the absence or presence of increasing concentrations of CLR01. Perturbation of the polymerization was observed only at 1,000 μM CLR01. The data are an average of three independent experiments and are shown as mean ± SEM.

In the absence of CLR01 or in the presence of up to 300 μM of the compound, the change in turbidity followed a typical sigmoidal curve, starting at 0.05-0.09 absorbance units (Figure 2). The absorbance remained unchanged for the first 10–15 minutes, which is a typical lag phase in this reaction, and then increased gradually up to ~60 min, at which point the rate of increase began to decline, and the reaction was followed for another 10 min. The only concentration at which significant modulation of the polymerization was observed was 1,000 μM (Figure 2, blue curve), i.e., at a tubulin:CLR01 concentration ratio ~1:55. At this high ratio, a high absorbance, 0.15, was observed immediately, followed by a slight gradual decline during the lag phase. Then, the absorbance began to increase for 30 min, followed by a slow decline for the rest of the experiment. One interpretation of these data is that at the high concentration used, 1,000 μM, binding of CLR01 to tubulin induced immediate self-assembly into irregular aggregates. Similar immediate induction of self-assembly was observed with 4 of the 9 amyloidogenic proteins tested by Sinha et al. [3], suggesting that this reaction occurs with some, but not all proteins. In all the cases studied by Sinha et al., these aggregates were non-amyloidogenic and non-toxic.

Presumably, following the immediate aggregation in the presence of 1,000 μM CLR01, the irregular tubulin aggregates observed at t = 0 partially disassembled as the polymerization reaction progressed, between 10–30 min. At that point, the high CLR01 concentration appeared to interfere with the polymerization reaction and the tubulin polymers gradually disassembled. Validation of this interpretation will require further investigation, yet it was not the focus of the current study.

The motivation for this experiment was to test whether the concentration of CLR01 needed to interfere with a controlled self-assembly process was substantially different from that required for modulation of aberrant self-assembly, which was found indeed to be the case. Most of the protein:CLR01 concentration ratios needed for inhibition of amyloidogenic protein aggregation were in the range 1:1–1:3 [3], compared to the 1:55 tubulin:CLR01 concentration ratio at which disruption of tubulin polymerization was observed. These results support the specificity of CLR01 for inhibition of aberrant aggregation as opposed to controlled polymerization.

### CLR01 safety

If CLR01 indeed operates by a process-specific mechanism and remodels the abnormal aggregation of amyloidogenic proteins at substantially lower concentrations than concentrations that would perturb normal physiological processes, one would expect the compound to have a high therapeutic index. To calculate the therapeutic index, a lethal dose must be reached. The *in vitro* data described above suggested that disruption of tubulin polymerization occurs at concentration ratios 20–50 times higher than those needed for inhibition of aggregation of amyloidogenic proteins. In addition, cell culture experiments indicated that CLR01 began to show toxicity at concentrations 1–3 orders of magnitude higher than those required for inhibition of toxicity by different amyloidogenic proteins [3,4]. The next rational step was to test the safety margin of CLR01 *in vivo*. Based on the *in vitro* and cell culture data, we expected that 100 mg/kg would be lethal to mice and therefore used it as the highest dose in our safety-evaluation experiments. We assessed the safety of CLR01 in 2-m old, male, WT mice either 24 h following a single IP injection of 10 or 100 mg/kg (acute administration) or after daily IP injection of 3 or 10 mg/kg for 30 days (chronic administration). Following euthanasia, serum was collected for chemical analysis and tissues were harvested for histopathology evaluation.

All CLR01-treated groups, except for the 100-mg/kg acute-administration group, behaved indistinguishably from control mice in terms of levels and type of activity and grooming. The administration of 100-mg/kg CLR01 caused obvious signs of distress immediately, which lasted for ~30 min following the injection. For most mice, activity level decreased and eyelids became droopy. Some of the mice exhibited arching of the back, sporadic gasping, lying down, dragging one leg, and twitching. These signs of distress diminished after the first 30 min, at which point the mice resumed grooming and sitting on hind legs. Some mice showed decreased activity and droopy eyelids for up to 2 h following the injection. No symptoms of severe toxicity, as defined by the UCLA DLAM veterinarians, were observed for any mice, including bruising, bleeding, pale mucous membranes or extremities, diarrhea, paralysis, tachypnea or dyspnea, or abdominal distension.

**Figure 3:**
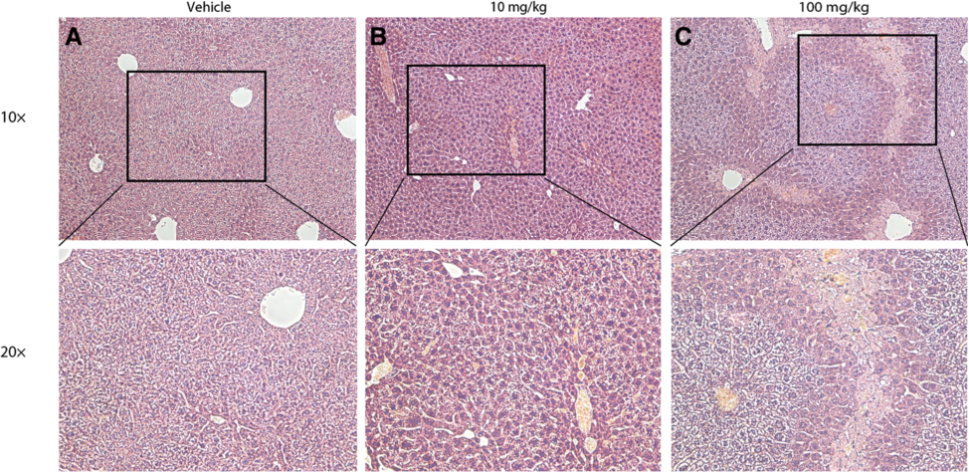
**Liver histopatholologic analysis of mice 24 h following a single IP injection of CLR01.** Hepatocytes from **A)** vehicle-treated, and **B)** 10-mg/kg-treated mice show moderate amounts of glycogen vacuolation. **C)** Zone-1 hepatocytes from 100-mg/kg-treated mice show glycogen vacuolation. Zone-2 hepatocytes are normal sized. Zone-3 hepatocytes are pale with granular eosinophilic cytoplasm and some nuclei show pyknosis.

Liver, kidney, spleen, heart, lung, and brain were collected for histopathology analysis. Tissue samples from heart, lung, spleen, and brain of all acutely CLR01-administered mice were indistinguishable from those of control mice. In all 100-mg/kg-dosed mice and one of eight 10-mg/kg-dosed mice of the acute-administration groups, liver degeneration and necrosis was detected in centrilobular and midlobular regions (Figure 3). Zonal nature of liver toxicity is common in drug-toxicity studies and was expected in the high-dose group.

The fact that all the mice in the high-dose group survived meant that the actual therapeutic index could not be calculated because contrary to our expectation, 100 mg/kg was under the lethal dose. However, we considered the observation of obvious liver toxicity at this high dose as sufficient for determining the maximal dose in future efficacy experiments and therefore did not treat mice with higher doses. Rather, we conducted next a 30-day, chronic-toxicity experiment in which mice were administered IP either 3 or 10 mg/kg/day of CLR01. Because one mouse of the eight used in the 10-mg/kg acute-administration group showed signs of liver toxicity, 10 mg/kg/day was chosen to be the highest dose in this experiment.

Heart, lung, spleen, and brain from both chronically CLR01-treated groups of mice were indistinguishable from vehicle-treated mice and were free of signs of malformation, degeneration, necrosis, or inflammation within normal variability among mice. A few mice in the 3-mg/kg group showed signs of mild-to-moderate multifocal extramedullary hematopoiesis in the liver. The consulting veterinary pathologist concluded that this was possibly immune-stimulated but not pathogenic. Mild pancreatitis also was observed in one of the mice showing liver hematopoiesis and one additional mouse in the 3-mg/kg group. In contrast, no signs of tissue pathology or liver necrosis were detected in any of the mice in the 10-mg/kg dosed group. Thus, it is unlikely that the hematopoiesis or inflammation found in the low-dose group were related to the CLR01 treatment.

Serum chemical analysis mainly consisted of tests of renal and liver function (Table 1). No significant differences were observed between the control and low-dose groups in either the acute-administration or chronic-administration experiments. The acute-administration, 100-mg/kg group showed significant increase in alanine aminotransferase, aspartate aminotransferase, and lactate dehydrogenase, and a significant decrease in cholesterol compared to both the control group and what is considered a normal range (UCLA DLAM, modified [23]). All of these changes are consistent with acute liver injury. Glucose levels were significantly lower in the 100-mg/kg acute-administration group than in the control group, but were within the normal range. Production of glucose is often the last function to be lost in liver damage. Other changes indicating liver damage, including changes in concentrations of albumin, alkaline phosphatase, or total bilirubin, were not observed. In the chronic-administration experiment, the only significant serum-chemistry difference observed was ~40% reduction in blood cholesterol in the 10-mg/kg group compared to the control group. The cholesterol level was within the normal range.

**Table 1:** Serum analysis

		**Acute, 24 h, single dose**			**Chronic, 30 day, daily dose**		
		**Control**	**10 mg/kg**	**100 mg/kg**	**Control**	**3 mg/kg**	**10 mg/kg**
		**N = 3**	**N = 8**	**N = 8**	**N = 9**	**N = 10**	**N = 9**
	**Normal range**	**Mean**	**Mean**	**Mean**	**Mean**	**Mean**	**Mean**
Alanine aminotransferase U/L	22–133	30.7	36.1	1282.9***	52.1	43.1	38.4
Aspartate aminotransferase U/L	46–221	63.3	89.5	565.3**	367.0	150.9	236.2
Albumin g/dL	2.6–5.4	2.2	2.3	1.9	2.8	2.8	2.7
Alkaline phosphatase U/L	16–200	107.7	126.1	101.5	98.1	104.4	106.1
Creatinine mg/dL	0.1–1.8	0.2	0.2	0.4	0.2	0.3	0.2
Total bilirubin mg/dL	0.3–0.7	0.2	0.2	0.3	0.3	0.2	0.2
Lactate dehydrogenase U/L	109–647	235.0	325.2	2439.8***	486.6	507.0	483.0
Blood urea nitrogen mg/dL	2–71	22.3	19.8	22.0	21.8	22.3	24.1
Cholesterol mg/dL	34–173	93.0	91.5	20.8**	92.3	80.9	56.8**
Total protein g/dL	4.6–7.3	4.0	3.7	3.6	4.8	4.7	4.6
Glucose mg/dL	60–133	286.0	297.8	104.3***	250.6	256.7	233.9

**p < 0.01. ***p < 0.001.

Values significantly different from the control group are highlighted in boldface.

### Pharmacokinetics of CLR01 *in vivo*

**Figure 4:**
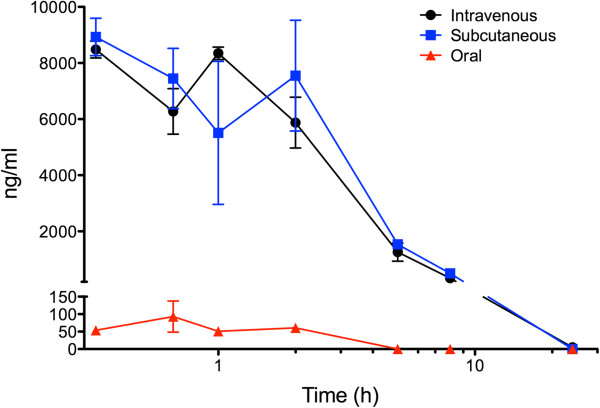
**CLR01 plasma concentration following different routes of administration.** The graph shows levels of CLR01 in plasma by intravenous (black line) or subcutaneous (blue line) injection at 1 mg/kg, or by oral gavage (red line) at 10 mg/kg over 24 h. Data are given as mean ± SD.

The plasma concentration of CLR01 was measured by LC-MS in 2-m old WT mice following administration by a SC or IV injection or by oral gavage. The SC bioavailability was found to be identical, within experimental error, to the IV administration, which was considered as 100% bioavailable (Figure 4). In both routes, ~30% of the administered dose was detected in the blood at the earliest time point measured – 20 min, and the plasma half-life was found to be ~2.5 h. Approximately 5% of the initial CLR01 levels were found in the plasma 8 h following either SC or IV administration. Oral bioavailability was negligible, suggesting that CLR01 gets metabolized in the gastrointestinal tract and/or does not pass from the gut to the blood.

Next, we asked what percentage of the administered CLR01 penetrates through the BBB and gets into the CNS. Our first attempt was to measure CLR01 in brain extracts using LC-MS. However, this proved to be difficult. Due to the multiple negative charges of CLR01, its partial protonation at physiologic pH, and the presence of various counter-ions in biological fluids, the MS signal splits into multiple peaks resulting in low signal-to-noise ratio. The difficulty to observe the CLR01 signal in brain extracts using LC-MS suggested that the concentration was low and detection would necessitate considerable optimization of the extraction and LC-MS methods, which would require substantial effort and high costs. Therefore, we decided to test first whether CLR01 could be found in the CNS by using a radiolabeled derivative of the compound.

As the permeability of the BBB has been shown to be dependent on age and morbidity, and in particular to be increased in AD [17] and in mouse models of AD [24,25], we assessed how age and disease progression affected the brain penetration of CLR01 by using WT and 3×Tg mice at three different ages. The 3×Tg model was chosen because it was used in a previous study, in which CLR01 was found to reduce AD-like pathology in the brain [5]. Mouse ages were chosen to correspond with: 1) a stage before Aβ burden and cognitive deficits are found at 2-m of age [14,26]; 2) a stage with mild-to-moderate plaque and tangle pathology but with observable memory deficits at 12-m of age [14,27]; and 3) a stage of abundant plaque and tangle pathology with consistent behavioral deficits at 22-m of age [28]. Mice were administered ^3^H-CLR01 IV, blood and brain were collected at time points between 0.5 - 72 h following CLR01 administration, and radioactivity levels were measured by liquid scintillation counting. Radioactivity is presented as CPM/g of brain or CPM/ml of blood.

**Figure 5:**
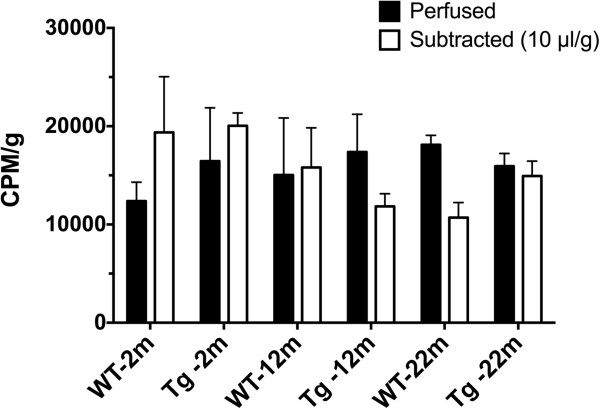
**Correction for radioactivity from residual blood in the brain.** Comparison of brain perfusion to remove residual blood with subtraction of calculated levels of blood radioactivity at 10 μl of blood per g of brain tissue. Data are given as mean ± SEM. The results were not significantly different and the subtraction method was used in the following experiments.

To correct for the radioactivity associated with blood-borne ^3^H-CLR01 in the brain vasculature, we performed both perfusion and subtraction analyses. In perfusion experiments, WT and 3×Tg mice at each of the three ages analyzed (n = 3 per group) were perfused with phosphate buffered saline following euthanasia. Perfusion lasted for either 5 min or until the liver changed color from a red to yellow, whichever was longer. In other experiments, mice were not perfused, but radioactivity associated with 10 μl of blood per g of brain [29,30] was calculated based on brain weight and blood radioactivity levels and subtracted from brain radioactivity levels. At 1 h post injection, perfusion-corrected brain values were statistically similar to subtraction-corrected brain values (Figure 5). Due to difficulties associated with the perfusion analysis, specifically liver color being used as an indirect readout of brain perfusion level, and because including a perfusion step could increase variability among experiments, the rest of the experiments utilized the subtraction method, which is a common practice in BBB-permeability studies [29,30].

**Figure 6:**
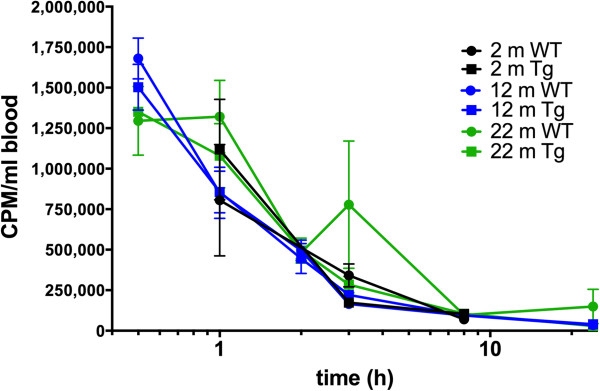
**Blood CLR01 levels in young, middle-aged, and old WT and 3×Tg mice.** CLR01 radioactivity levels, measured by scintillation counting, are given as CPM per ml of blood for the six mouse groups between 0.5 - 24 h. At 8 h post administration CLR01 levels drop to 5 - 10% of values observed at 0.5 h. Data are given as mean ± SEM.

At 0.5 h following injection, blood radioactivity levels in 12-m old mice were 39 ± 13% and 40 ± 6% of the injected levels, for WT and 3×Tg mice, respectively. These values were in agreement with the CLR01 concentration levels detected in plasma by LC-MS. About 5 - 10% of the radioactivity observed at time 0.5 h remained in the blood after 8 h (Figure 6).

**Figure 7:**
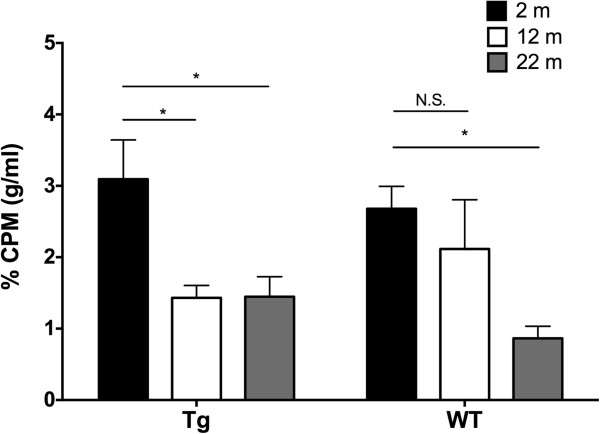
**Percent brain penetration of CLR01 at 1 h.** Percent of CLR01 radioactivity per g of brain was calculated as a function of blood radioactivity levels per ml at 1 h following IV administration of CLR01 ((CPM/g)/(CPM/ml) × 100). Data are given as mean ± SEM. *p < 0.05.

Brain-radioactivity levels, calculated as a percentage of blood-radioactivity levels (CPM/g)/(CPM/ml) at 1 h following the injection ranged from 0.86–3.09% depending on age and genotype (WT versus 3×Tg, Figure 7). Analysis of brain penetration levels at 1 h by absence or presence of AD transgenes and by age showed a statistically significant effect of age but not of genotype. Interestingly however, 2-m old 3×Tg mice significantly differed from 12-m and 24-m old 3×Tg mice (2-m: 3.09 ± 0.55%; 12-m: 1.43 ± 0.17%; 24-m: 1.45 ± 0.28%; *p* < 0.05), whereas in the WT group, the only significant difference was between the 2-m and 24-m old mice (2-m: 2.68 ± 0.31%; 12-m: 2.11 ± 0.69%; 24-m: 0.86 ± 0.17%; *p* < 0.05). This suggests that changes in BBB permeability occur earlier and more sharply in 3×Tg mice compared to WT mice.

**Figure 8:**
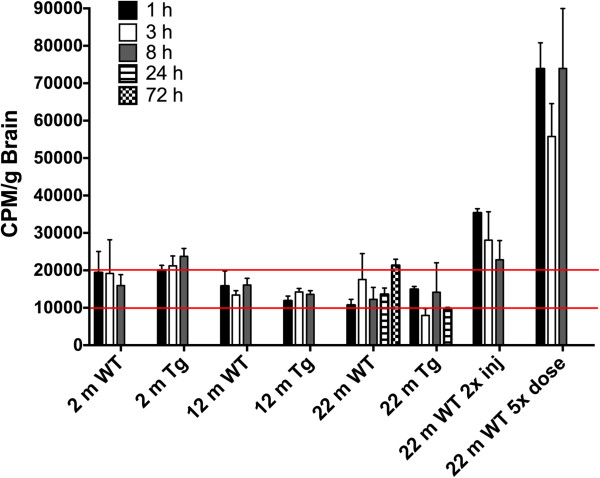
**Brain CLR01 levels in young, middle-aged, and old WT and 3×Tg mice.** CLR01 radioactivity levels are given per g of brain. Most group × time combinations fall between 10,000 - 20,000 CPM/g (marked with red lines). Double injection studies in aged WT mice show on average double the radioactivity levels of single injection group, 22 m WT. Aged WT mice dosed with 5× the amount of CLR01, show on average 5-times the radioactivity levels of the 1× group, 22 m WT. Data are given as mean ± SEM.

Surprisingly, although blood radioactivity levels declined rapidly (Figure 6), the radioactivity levels measured in the brain did not change significantly up to 72 h post-injection (Figure 8). Brain radioactivity levels were insensitive to genotype or time after injection and thus the 24-h time point was assessed only in the 22-m old mice (both 3×Tg and WT) and the 72-h time point was assessed only in the 22-m old WT mice. Differences were statistically insignificant and within experimental error.

To explore further the mechanics of CLR01 transport across the BBB, we asked whether the transport system was saturated. To answer this question, we injected 5-times the amount of total CLR01, keeping the ratio of ^3^H-CLR01:CLR01 at 1:9, into 22-m old WT mice. This experiment resulted on average, in 5-times the absolute amount of radioactivity detected in the brain. The percentage of brain penetration at 1 h following the injection did not change significantly (1× CLR01 brain penetration: 0.86 ± 0.30% of blood; 5× CLR01 brain penetration: 0.97 ± 0.28% of blood; Figure 8). This result suggests that the transport mechanism, whether active or passive, is concentration-dependent because there was an increase in the absolute value but not the relative value of CLR01 entering the brain.

To begin to explore whether additional dosing would increase the effective CLR01 concentration in the brain, we injected 22-m old WT mice twice over two equal time intervals and compared brain levels to mice that received one injection. On average, over the 1-, 3-, and 8-h time points measured, the amount of radioactivity found in the brain following the double-injection was twice the amount measured following the single-injection protocol (1 h: 3.3× compared to one injection, 3 h: 1.6×, 8 h: 1.9×; Figure 8). These data suggest that upon continuous dosing, as with the SC osmotic mini-pumps used in our previous efficacy study [5], CLR01 could reach sufficiently high brain concentration levels to inhibit Aβ aggregation even though the dose was relatively low — 40 μg/kg/day – when brain penetration levels are taken into account (see Discussion).

### *In vitro* catabolism of CLR01

**Figure 9:**
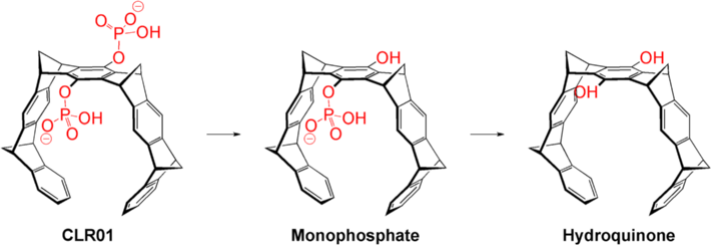
**CLR01 dephosphorylation.** Molecular structure of theoretical successive CLR01 dephosphorylations at the bridgehead to monophosphate and then to hydroquinone.

The BBB permeability experiments described above used radioactivity as an indirect readout of CLR01 concentration levels, which could have reflected the parent compound, CLR01 itself, or its metabolites. The question of the source of radioactivity seemed particularly important in view of the surprising persistence of radioactivity attributed to CLR01 in the brain. The most likely metabolism of CLR01 is cleavage of one or both phosphate groups resulting in monophosphate and hydroquinone derivatives, respectively (Figure 9). Each such dephosphorylation would decrease the polarity of the compound and increase its potential partition into the lipophilic brain parenchyma environment relative to the blood. In particular, the hydroquinone product is insoluble in aqueous solutions, in contrast to CLR01 and its monophosphate metabolite, which are soluble at millimolar concentrations. Thus, double dephosphorylation could result in precipitation and accumulation of the hydroquinone in the brain, potentially leading to misinterpretation of the BBB permeability data. Complete analysis of CLR01 metabolism in the brain was beyond the scope of the study described here. However, to evaluate the potential for dephosphorylation, we incubated CLR01 *in vitro* with ALP or brain extracts and measured the release of inorganic phosphate.

ALP is a widely distributed plasma enzyme found in many tissues which can be released into body fluids [31]. The enzyme received its name because it shows optimal activity at pH ~9. There are four isoforms of ALP: intestinal, placental, germ cell, and tissue non-specific. All four isoforms are non-specific enzymes that catalyze the hydrolysis of a wide range of phosphate esters [32]. Tissue non-specific ALP concentration levels increase in both brain and plasma of patients with familial or sporadic AD relative to age-matched healthy individuals [33], possibly as a compensatory mechanism because the enzyme catalyzes tau dephosphorylation [34].

Because of its promiscuous hydrolysis activity, we tested whether calf intestinal ALP catalyzed CLR01 dephosphorylation by incubating the molecular tweezer with ALP and comparing the amount of inorganic phosphate released to a standard curve obtained by incubating ALP with increasing concentrations of a common substrate, p-nitrophenylphosphate. This standard curve had a detection sensitivity limit of 5 nmol. Incubation of up to 100 nmol CLR01 with ALP resulted in undetectable levels of inorganic phosphate, suggesting that despite its promiscuity, ALP did not catalyze dephosphorylation of CLR01.

To test whether CLR01 dephosphorylation might be catalyzed by brain phosphatases other than ALP, we incubated 50 nmol CLR01 with 1.5 mg of mouse-brain homogenate. The brain homogenate dephosphorylated the positive control substrate, p-nitrophenylphosphate, at 99 - 130% of the activity of 0.8 enzymatic units of ALP. In contrast, similarly to the reaction with ALP, no release of inorganic phosphate was detected when the brain homogenates were incubated with CLR01 under the same conditions. Based on these results, dephosphorylation of CLR01 likely did not happen in our BBB permeability experiments and the radioactivity measured in mouse brains plausibly reflected CLR01 itself.

## Discussion

Recently, we have reported that CLR01, an inhibitor of aberrant assembly and toxicity of amyloidogenic proteins [3], protected primary neurons from Aβ-induced decrease in synaptic spine density, basal synaptic activity, and long-term potentiation [5]. In addition, CLR01 treatment of 15-m old 3×Tg mice with 40 μg/kg/day CLR01 for 28 days resulted in decreased AD-related brain pathology, including amyloid plaques, neurofibrillary tangles, and microglia levels [5]. In a mouse model of familial amyloidotic polyneuropathy, CLR01 significantly decreased transthyretin deposition and associated pathological markers, including ER stress, oxidative stress, and apoptosis [15]. Following up on these promising efficacy data, here, we explored the putative process-specific mechanism of CLR01, its safety margin in mice, its BBB permeability and how it might be affected by age and disease, and the most likely route of CLR01 metabolism.

As stated above, no signs of toxicity have been observed in *in vivo* efficacy studies. Towards determining optimal dosing for subsequent studies, we sought to find out the lethal dose, which would provide an upper limit for future dosing decisions. Effectively, we found that our highest acute dose of 100 mg/kg was not lethal but did elicit obvious behavioral signs of distress and liver damage (Table 1). Thus, chronic dosing at this concentration could lead to mortality. Importantly, we found that high doses of CLR01 had no effect on brain, heart, lung, spleen, or kidney. Liver injury, found by both histology and serum analysis, was the main indicator of acute toxicity. These data will be used to direct monitoring for potential toxicity in future studies using higher doses than those used previously and potentially using species other than mouse.

In the chronic-administration experiment, the only meaningful finding was a decrease in cholesterol levels, which were still within the normal range, in the 10-mg/kg/day group (Table 1). This was an unexpected effect of CLR01 treatment, and may be of interest for further exploration especially for dual treatment of AD as high cholesterol in middle age is associated with increased risk for AD [35,36]. Importantly, the chronically administered dose of 10 mg/kg/day is 250-times higher than the efficacious dose of 40 μg/kg/day used in the AD model [5] and thus provides a large safety margin. In support of this conclusion, concentrations up to 300 μM did not significantly affect the polymerization of tubulin *in vitro* (Figure 2), suggesting that CLR01 does not inhibit physiologic protein assembly unless the concentrations used are substantially higher than those needed for therapeutic effects.

Several previous observations support the safety of CLR01. As mentioned above, *in vivo*, CLR01 decreased significantly brain Aβ deposition without interfering with APP processing [5], and inhibited α-synuclein aggregation but not its ubiquitination, which requires free Lys residues [4]. In addition, when CLR01 was tested *in vitro* as an inhibitor of enzymatic activity, the CLR01:enzyme concentration ratio needed for inhibition was orders of magnitude higher than the ratios needed for inhibition of abnormal protein aggregation. Thus, CLR01 inhibited alcohol dehydrogenase (ADH) activity with IC_50_ = 180 μM at ADH concentration of 208 nM [7]. Thus, the CLR01:ADH ratio needed for inhibition was 865:1. In another study, CLR01 was tested as an inhibitor of Poly [ADP-ribose] polymerase 1 (PARP-1). Inhibition was found with an IC_50_ of 3.3 μM at an enzyme concentration of 2.3 nM (T. Schrader, unpublished results). The ratio in this case was 1435:1. These findings support the proposed process-specific mechanism and the development of molecular tweezers in general, and CLR01 in particular, towards initiation of clinical trials.

It is important to note that animal dose should not be extrapolated to a human equivalent dose by conversion of body weight, but rather by normalization to body surface area [37]. This method correlates well with several parameters of biology, including oxygen utilization, caloric expenditure, basal metabolism, blood volume, circulating plasma proteins, and renal function [38]. An extrapolation using the body surface area suggests that a dosing window between 0.04 – 10 mg/kg/day in mice corresponds to 0.003 - 0.81 mg/kg/day in humans.

Many of the properties of the BBB that determine the extent to which drugs are taken up by the brain are known to be altered in AD, such as disruption of tight junctions, decreased CSF reabsorption, decreased cerebral blood flow, and decreased efflux pump activity [17]. Similar BBB compromise has been reported in animal models of AD [24,25], thus, we set out to explore the differences in CLR01 brain penetration in both WT and the 3×Tg mouse model of AD. Because many of these properties, such as CSF reabsorption and BBB disruption, are not simply binary, we chose animals at three different ages, from 2 - 22-m, which correlate with different stages of disease progression, to evaluate the effect of age and disease on drug uptake. Using ^3^H-labeled CLR01, we found brain penetration levels between 1 - 3% in the different ages, whereas the absence or presence of AD transgenes had little effect on CLR01 uptake into the brain (Figure 7). There was no statistically significant interaction between age and presence of AD transgenes. However, we did find that 2-m old 3×Tg mice differed significantly from 12-m and 22-m old 3×Tg mice. In comparison, in the WT group, the only significant difference was between the 2-m and 22-m old mice. These data suggest that the presence of the AD-related transgenes expedites the disintegration of the BBB and thus increases the brain penetration of CLR01 by a small, yet potentially meaningful, amount. Unexpectedly, we found higher penetration of CLR01 in the brains of the younger mice. One possible explanation of these findings, assuming that CLR01 enters the brain by a passive transport mechanism, is that the increased BBB permeability observed at old age results in faster leakage of CLR01 out of the brain than in the young mice. Alternatively, CLR01 may be taken up by a serendipitous active transport system that is more efficient in younger, than in older mice.

The observation that brain radioactivity did not decline with time (Figure 8) was peculiar. Linear regression analysis of the values between 1–72 h for the 22-m old WT mice resulted in a slope that was not significantly different from zero. This unexpected behavior raised a concern for a systematic error producing these data. However, both the double-injection-, and the 5×-dose-experiments showed a linear increase in brain radioactivity, suggesting that the radioactivity measured in the brain reflected *bona fide* uptake of CLR01 through the BBB. Another concern was that the radioactivity measured in the brain actually came from residual blood that was not accounted for by either perfusion or subtraction of the expected values. However, the observations that blood CLR01 or ^3^H-CLR01 decreased to ~5% of the starting values by 8 h (Figures 4 and 6) without a correlating decrease in brain radioactivity, which remained steady over that same period, indicated that the radioactivity measured in the brain was not related to residual blood levels. In addition, actual sample counts (≥500 CPM) were well above the minimum sensitivity of the liquid scintillation counting system (background was < 150 CPM). Thus, the radioactivity measured in the brain reflected the actual ^3^H-CLR01 levels that penetrated the brain.

The observation that CLR01 penetration levels were consistent among groups and persistent over time suggests that CLR01 enters the brain and accumulates with parameters that are age-specific, as age was found to be a significant determinant of BBB penetration (Figure 7). One possible mechanism by which CLR01 passes across the BBB is by binding to Lys residues on receptors that span the membrane or get endocytosed. An analysis of the amino acid sequence of four major human cellular receptors involved in transferring cargo across the BBB – transferrin, low density lipoprotein receptor-related protein 1, glucose transporter 1, and large neutral amino acid transporter – reveals that Lys makes up 3.3 - 6.6% of their sequences. If these Lys residues are exposed and are positioned within the receptor’s channel, or get endocytosed upon ligand binding, they may allow CLR01 to “hitchhike” its way across the BBB through its labile binding to these receptors and potentially through the transport of the natural cargo.

An important question is whether the mechanism of action of CLR01 *in vivo* is similar to, or different from, its mechanism *in vitro*. Though complete characterization of the *in vivo* mechanism is difficult to achieve, an important question is whether the stoichiometry of the molecular tweezer and its target proteins is similar *in vitro* and *in vivo*. Based on experiments using SC pumps in which 0.7% of the administered CLR01 was in the blood at steady-state (Lopes DHJ, Attar A, Du Z, McDaniel K, Dutt S, Bravo-Rodriguez K, Ramirez-Anguita JM, Sancez-Garcia E, Klärner F-G, Wang C, *Schrader T, Bitan G*: The molecular tweezer CLR01 inhibits islet amyloid polypeptide assembly and toxicity via an unexpected mechanism, submitted), the brain penetration of ~2% of blood levels found here, and the efficacy studies in the 15-m old 3×Tg mice using a 40-μg/kg/day dose [5], we estimate that ~200 fmol of CLR01 enter the brain per day. A literature search for brain concentration levels of Aβ40 and Aβ42 resulted in reported values from zero to a maximum of 280 fmol/mg brain [14]. The masses of the mouse brains used in our studies were ~0.5 g. Thus, a total of ~140 fmol Aβ may be found at a given point in 13-m old 3×Tg mice [14]. Upon accumulation of CLR01 in the brain, as we observed in the double-injection experiment, the concentration levels of CLR01 entering the brain at a 40-μg/kg/day dose and of Aβ are expected to be on the same order of magnitude, specifically, in the range of hundreds of fmols. This is not to suggest that CLR01 does not interact with all exposed Lys residues on any protein. It likely does. However, whereas the high on-off rate of CLR01 binding to Lys residues [9] is unlikely to disrupt stable protein structures significantly, because self-association of amyloidogenic proteins depends on the improbable formation of a nucleus comprising multiple monomers, presumably binding of CLR01 to a small percentage of the monomers would be sufficient for disruption of nucleus formation. The same rationale is applicable to formation of metastable toxic oligomers, which are made of multiple monomers. Thus, substoichiometric concentrations of CLR01 relative to its target protein are expected to be sufficient for producing a beneficial effect. The analysis outlined above suggests that the intracranial Aβ:CLR01 stoichiometry achieved in our *in vivo* study, in which we found substantial decrease in AD-like pathology [5], was similar to the stoichiometry in *in vitro* and cell culture experiments [3] providing strong support for the putative mechanism of action of CLR01.

The estimate of 200 fmol of CLR01 entering the brain per day upon administration of 40 μg/kg/day [5] is a conservative one, when considering two additional factors. First, the levels of CLR01 detected in the plasma following an IV injection, which is considered 100% bioavailable, were about 30% of amount injected. Thus, the amount detected may reflect the limitation of the detection method and the CLR01 actual concentration in the blood may be higher. Second, the cerebrovascular volume of the 3×Tg mice at 11-m of age has been shown to be 26% lower than that of non-transgenic littermates, potentially due to cerebrovascular amyloid deposition [39]. We did not take this difference into account in our correction for cerebral blood when calculating brain radioactivity and thus might have biased our data to reflect lower radioactivity in the older 3×Tg mice than actual values. Taking these potential biases into account lends additional support to the suggested mechanism of action of CLR01 *in vivo*.

An important factor for development of CLR01 and/or other molecular tweezer derivatives as therapeutic drugs is identifying the active pharmaceutical ingredient. *In vitro* data suggest that binding of CLR01 itself to free Lys residues is what modulates the self-assembly of amyloidogenic proteins into non-amyloidogenic, non-toxic species. However, *in vivo*, CLR01 may be metabolized in currently unknown ways and the active pharmaceutical ingredient may be a metabolite. To examine potential CLR01 metabolism, previously, we tested the stability of the compound in mouse and human, plasma and liver microsomes and found 100% stability in all preparations [5]. To explore the question of stability and potential metabolism further, here, we hypothesized that the phosphate groups would be the most likely targets of metabolism and therefore asked whether they were substrates for dephosphorylation by ALP or other brain phosphatases. The question was of particular importance in view of the reported increase in ALP concentration in both brain and plasma of patients with AD relative to healthy individuals [33]. We tested the potential dephosphorylation of CLR01 under stringent conditions of excess ALP in buffer and did not find release of inorganic phosphate upon incubation of CLR01 with either the purified phosphatase or the brain extracts. A plausible explanation for the observed stability of CLR01’s phosphate groups to enzymatic dephosphorylation is the rigid structure of the hydrocarbon backbone of the compound (Figures 1 and 9), which likely prevents its accommodation in the active sites of phosphatases.

Process-specific modulation of amyloid protein assembly is a useful approach that can be adopted for a multitude of amyloidoses. The beneficial therapeutic effects of CLR01 have been demonstrated in mouse models of AD and familial amyloidotic polyneuropathy, and a zebrafish model of Parkinson’s disease [11]. Here, we found a favorable safety profile and small yet persistent brain penetration – a formidable starting point for future formal development of CLR01 towards human therapy.

## Conclusion

A single dose of CLR01, at 2,500-fold the efficacious dose, induced behavioral distress and liver injury, but did not result in mortality. Daily dosing at 250-fold the efficacious dose did not result in any signs of behavioral, serological, or pathological toxicity. *In vitro* evaluation of CLR01’s influence on physiologically normal protein assembly did not show disruption until 55-fold excess of CLR01 was used. These results indicate a high safety margin for CLR01. Brain penetration of CLR01 was observed to be ~2% of blood levels depending on age, yet it was persistent for 3 days. These data were used to verify that sufficient levels of CLR01 are present in the brain for the putative mechanism of action.

## Abbreviations

3×Tg: Triple transgenic; α-syn: α-synuclein; AD: Alzheimer’s disease; APP: Amyloid β-protein precursor; ALP: Alkaline phosphatase; BBB: Blood–brain barrier; CNS: Central nervous system; CPM: Counts per minute; DLAM: Division of Laboratory Animal Medicine; IP: Intraperitoneal; IV: Intravenous; LC-MS: Liquid chromatography-mass spectrometry; SC: Subcutaneous; SEM: Standard error of the mean; WT: Wild type.

## Competing interests

AA and WTCC have no competing interests to declare. FGK, TS, and GB are co-authors and co-inventors of International Patent Application No. PCT/US2010/026419, USA Patent Application No. 13/203,962, European Patent Application 10 708 075.6. GB is a Director and a Co-Founder of Clear Therapeutics, Inc.

## Authors’ contributions

AA and GB conceived and designed the research, analyzed the data, and wrote the manuscript. AA, WTCC, and GB performed the research. FGK and TS contributed reagents. All authors read and approved the final manuscript.

## Pre-publication history

The pre-publication history for this paper can be accessed here:

http://www.biomedcentral.com/2050-6511/15/23/prepub
